# Supplementary material for: Identification of photoperiod-induced specific miRNAs in the adrenal glands of Sunite sheep (Ovis aries)
Source: Front Vet Sci. 2022 Jul 22;9:888207. doi: 10.3389/fvets.2022.888207 (PMC9354845; doi:10.3389/fvets.2022.888207)
Supplement: Supplementary file 1 [file Data_Sheet_1.ZIP › Supplementary file/miRDeep2 file.zip!/miRDeep2 file/Know_Structure/SP42A1/oar-let-7f.pdf]

miRBase precursor : oar-let-7f
Total read count : 421082
oar-let-7f read count : 421000
remaining reads : 82

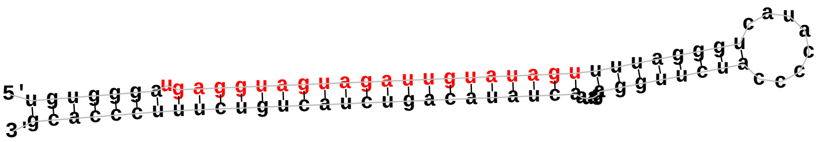

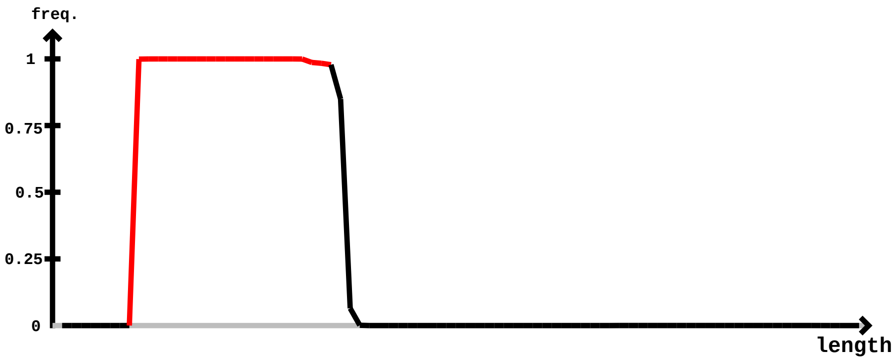

oar-let-7f

```
5'- uguggga ugagguaguagauuguauagu uuuagggucauaccccaucuuggagauaacuauacagucuacugucuuucccacg   -3'    exp
    .((((((.((((((((((((((((((((((((((((........))))))))....)))))))))))))))))))))))))).   reads   mm   sample
    ......augagguaguagauuguau..........................................................   1       0    seq
    ......augagguaguagauuguauagu.......................................................   5       0    seq
    ......Gugagguaguagauuguauaguu......................................................   2       1    seq
    ......augagguaguagauuguauaguu......................................................   22      0    seq
    ......augagguaguagauuguauaguuu.....................................................   1       0    seq
    .......ugaAguaguagauugua...........................................................   2       1    seq
    .......ugagguaguagauuguU...........................................................   2       1    seq
    .......ugagguaguagauugua...........................................................   175     0    seq
    .......ugagguagGagauugua...........................................................   1       1    seq
    .......ugagguaguagauuguau..........................................................   5337    0    seq
    .......ugaAguaguagauuguau..........................................................   26      1    seq
    .......ugagguaguagauuguUu..........................................................   10      1    seq
    .......ugagguaguagauugAau..........................................................   1       1    seq
    .......ugagguaguUgauuguau..........................................................   3       1    seq
    .......ugagguaguagauugAaua.........................................................   1       1    seq
    .......ugagguaguagauuguaua.........................................................   1307    0    seq
    .......ugagguaguagauuguauag........................................................   2256    0    seq
    .......ugagguaguagauuguauagu.......................................................   54172   0    seq
    .......ugagguaguagauugAauagu.......................................................   10      1    seq
    .......ugagguaguagauuguauaguu......................................................   330240  0    seq
    .......ugagguaguagauuguauaguuu.....................................................   25689   0    seq
    .......ugagguaguagauuguauaguuG.....................................................   1049    1    seq
    .......ugagguaguagauuguauaguuuu....................................................   300     0    seq
    .......ugagguaguagauuguauaguuGu....................................................   105     1    seq
    .......ugagguaguagauuguauaguuuua...................................................   5       0    seq
    ........gagguaguagauuguau..........................................................   4       0    seq
    ........gagguaguagauuguauagu.......................................................   27      0    seq
    ........gagguaguagauuguauaguu......................................................   188     0    seq
    ........gagguaguagauuguauaguuu.....................................................   14      0    seq
    ........gagguaguagauuguauaguuG.....................................................   2       1    seq
    .........agguaguagauuguauaguu......................................................   4       0    seq
    .........agguaguagauuguauaguuG.....................................................   1       1    seq
    .........agguaguagauuguauaguuu.....................................................   1       0    seq
    .........agguaguagauuguauaguuGu....................................................   33      1    seq
    ..........gguaguagauuguauaguu......................................................   2       0    seq
```

oar-let-7f

```
uguggga ugagguaguagauuguauagu uuuagggucauaccccaucuuggagauaacuauacagucuacugucuuucccacg
...........guaguagauuguauaguu.....................................................    1    0    seq
............uaguagauuguauaguu.....................................................    1    0    seq
.............................uuagggucauaccccaucuuggaga.............................    1    0    seq
.............................uuagggucauaccccaucuuggagaua...........................    1    0    seq
........................................................acuauacagucuacugucuuuc.....    1    0    seq
.........................................................cuauacagucuacugucuuu......    5    0    seq
.........................................................cuauacagucuacugucuuuc.....    12   0    seq
.........................................................cuauacagucuacugucuuucc....    62   0    seq
```
